# Remind Me, My Memory Is All Shook Up

**DOI:** 10.1523/ENEURO.0379-22.2022

**Published:** 2022-10-13

**Authors:** Laura Dovek

**Affiliations:** 1 Department of Molecular, Cell and Systems Biology, University of California, Riverside, Riverside, California 92521; 2 Biomedical Sciences Graduate Program, University of California, Riverside, Riverside, California 92521

Our brains synthesize vast and varied sensory inputs to represent an event or place that can later be recalled freely. The term “engram” refers to a group of neurons activated in association with a memory or event, suggesting that if one were to activate a subset of cells in the ensemble, one would trigger the recall of a memory. The medial temporal lobes, including the hippocampus, are often studied as the primary region of spatial engram formation (Josselyn and Tonegawa, 2020). However, newly formed spatial memory representations are incredibly transient. Following encoding, consolidation from the hippocampus to related cortical structures is critical for stable memory retention (Wirt and Hyman, 2017). What happens when this process of generating stable long-term memories is disrupted? Neuronal hyperactivity, seen in seizures, potentially corrupts existing engrams and is often associated with retrograde amnesia in humans. Retrograde amnesia, or forgetting recently formed memories, could be because of disruptions in memories being transferred from the hippocampus to cortical structures. Circuits in the hippocampal dentate gyrus (DG), proposed to support memory consolidation, act as an alternative route for seizure spread from the hippocampus to the neocortex, raising the possibility that unstructured activity in seizures may degrade the structured process of memory formation (Dabrowska et al., 2019). This intriguing possibility is explored in the study by Naik et al. (2022).

The recent article builds on their previous study (Naik et al., 2021), where activity reporter transgenic mice were used to evaluate the interaction between spatial memory and acute seizures in hippocampal CA1 neurons. Their 2021 study adopted a spatial learning task where mice improved in performance over time. Following chemically induced seizure, they observed significant deficits in task performance. However, mice that endured acute seizures learned the task with additional trials, implying they experienced retrograde amnesia and not long-term memory deficits. The study identified a striking overlap between the CA1 neuronal ensembles activated by the memory task and seizures, suggesting that the conflation of activity patterns of the spatial engram with those of seizures may underlie retrograde amnesia.

The current study (Naik et al., 2022) adopted a holistic approach to analyze the intersection between circuits activated during memory and seizure. They examine (1) whether circuits outside of the hippocampal CA1 show overlapping neuronal activation during memory and seizure, indicating a role in retrograde amnesia; and (2) whether specific brain regions involved in spatial memory processing are preferentially activated by seizures. Using transgenic TRAP2 mice to selectively and permanently “tag” neurons activated during a T-maze spatial memory task, they examine the recruitment of hippocampal-related structures, including regions involved in spatial memory. TRAP2 mice are induced, by tamoxifen injection, to express a fluorescent reporter under the control of the promoter for c-Fos, an immediate early gene expressed in active neurons. By inducing labeling of neurons activated after days 1–3 of T-maze trials, they identify that retrosplenial cortex (RSC), medial prefrontal cortex (mPFC) and DG showed the most robust activation on day 2. Absences of preferential recruitment of periventricular thalamus (PVT), which does not support spatial memory, provided a clever control. TRAP2 mice lacking GLUA1, an AMPA receptor subunit required for spatial memory, showed poor task performance and low neuronal activation, demonstrating reliable and optimal tagging of memory-related circuits. Interestingly, pentylenetetrazole (PTZ)-induced seizures activated the same networks as spatial memory, although the activation of PVT was not evaluated.

Having established that they could tag memory-related circuits and that similar networks were activated by PTZ, they tagged neurons in control and T-maze-trained TRAP2 mice followed by acute seizure induction a week later to test the overlap between memory and seizure circuits. They found that colocalization between neurons tagged during the T-maze task and seizure-activated neurons, immunolabeled with the immediate early gene ARC, was significantly greater than that expected by chance or observed in controls. The enhanced overlap occurred in the RSC and mPFC but not in the PVT, demonstrating a strong intersection between neurons activated during spatial memory and seizures. These findings suggest that seizures contribute to retrograde amnesia by involving memory ensembles in abnormal, unrelated activity. Indeed, neurons with higher intrinsic or circuit-driven excitability are likely to be more active in engrams (Cai et al., 2016) and to be recruited in seizures, suggesting that cellular and circuit factors may underlie the overlap. Active neurons being jointly recruited to memory ensembles and seizures supports the theory that destabilizing engrams results in retrograde amnesia. These findings advance our understanding of seizure-induced memory impairments that could be of therapeutic relevance.

The study highlights how the TRAP2 strategy can be elegantly adapted to label and analyze processes at the intersection of transient phenomenon of memory formation and seizures. However, the potential issues posed by the longer window for tagging “active” neurons rather than the tasks and needs for habituation for animal handling and injection to minimize task-independent neuronal activation need to be considered. Although cFOS expression has been shown to be greater than that of ARC in certain tasks (Heroux et al., 2018), the overlap between cFOS tagging and ARC staining observed in several regions indicates robustness of the approach. However, it is possible that the overlap in the sparsely active DG may have been underestimated by ARC staining. It is notable that the distributed brain structures often associated with long-term memory storage were tagged during the behavior task, suggesting engagement of the memory consolidation process or the recall of consolidated memories. These studies also open the question of whether neurons activated as part of engrams in interconnected regions share properties that make them susceptible to preferential recruitment in seizures: are they inherently more excitable cells? Do they share circuit architectures that enable them to activate in cohorts? Future advances in the methods used to tag cells during the consolidation process could open therapeutic avenues to disrupt their recruitment in seizures and protect memories.

Currently, we have an incomplete understanding of the pathophysiology of seizure disorders and thus the lack of proper therapeutics. By demonstrating that seizures and memory occupy the same brain regions, potentially perturbing hippocampal–cortical interactions involved in consolidation, this study moves us a step closer to fully understanding seizure-related memory issues. The global approach of the study is notable and necessary to fully understand how seizures disrupt learning and memory circuits, resulting in retrograde amnesia.

## Synthesis

Reviewing Editor: Viji Santhakumar, University of California Riverside
